# A conceptual re-evaluation of reproductive coercion: centring intent, fear and control

**DOI:** 10.1186/s12978-021-01143-6

**Published:** 2021-04-27

**Authors:** Laura Tarzia, Kelsey Hegarty

**Affiliations:** 1 Department of General Practice, The University of Melbourne, Level 2, 780 Elizabeth Street, Melbourne, VIC 3010 Australia; 2 Centre for Family Violence Prevention, The Royal Women’s Hospital, Parkville, VIC Australia

**Keywords:** Reproductive coercion, Intimate partner violence, Sexual violence, Reproductive autonomy, Women, Family violence

## Abstract

**Background:**

Reproductive coercion and abuse (RCA) is a hidden form of violence against women. It includes behaviours intended to control or dictate a woman’s reproductive autonomy, for the purpose of either preventing or promoting pregnancy.

**Main text:**

In this commentary, we argue that there is a lack of conceptual clarity around RCA that is a barrier to developing a robust evidence base. Furthermore, we suggest that there is a poor understanding of the way that RCA intersects with other types of violence (intimate partner violence; sexual violence) and—as a result—inconsistent definition and measurement in research and healthcare practice. To address this, we propose a new way of understanding RCA that centres perpetrator intent and the presence of fear and/or control. Recommendations for future research are also discussed.

**Conclusion:**

We suggest that IPV and SV are the *mechanisms* through which RCA is perpetrated. In other words, RCA cannot exist without some other form of co-occurring violence in a relationship. This has important implications for research, policy and healthcare practice including for screening and identification of women in reproductive healthcare settings.

## Background

Reproductive coercion and abuse (RCA), first defined as simply “reproductive coercion” by Elizabeth Miller and colleagues in 2010, refers to any deliberate attempt to influence or control a person’s reproductive choices [1] or interfere with their reproductive autonomy. It is typically perpetrated against women by a male intimate partner [2], although other family members can also be participants or instigators [3]. RCA is commonly understood to take three main forms: pregnancy coercion (where a woman is pressured or forced to become pregnant against her will); contraceptive sabotage (deliberately damaging, hiding, or otherwise interfering with birth control); and controlling the outcome of a pregnancy (forcing a woman to terminate or continue a pregnancy against her will) [2]. Extant literature suggests associations between RCA and intimate partner violence (IPV) [3–6], unwanted pregnancies [2, 7], poor mental health [8], decreased contraceptive self-efficacy [9, 10], and increased risk of sexually transmitted infections [11]. These negative health outcomes have led to a recent rise in media interest in the issue. We argue in this commentary, however, that we have seen a lack of definitional and conceptual clarity around RCA, leading to inconsistency across prevalence data, a poor understanding of the risk factors, and difficulties in demonstrating the effectiveness of interventions in health settings. RCA research remains in its infancy; it is therefore an opportune time to pause and re-evaluate the state of the knowledge, as well as to reflect on the best way forward in this emerging field.

Our perspective in writing this commentary is as health and violence researchers working in a university setting in a high-income country. One of us (KH) has a clinical background as a general practitioner and LT is an applied sociologist. Our arguments and their implications for research and practice are thus shaped by the assumptions that: (1) violence against women is a major public health issue; (2) health systems are well-placed to identify and respond to violence against women and (3) a solid theoretical and empirical evidence base is critical for informing the development and implementation of interventions in health settings. We recognise and welcome discussion of conceptual issues relating to RCA within other disciplines such as philosophy and gender studies; however, our purpose is to define and discuss RCA insofar as it affects healthcare practice and outcomes for women.

### What do we know about RCA?

It is not our intention here to review the growing body of literature on RCA. This has been covered by several comprehensive systematic reviews [2, 7, 12, 13] which address the prevalence and types of reproductive coercion and its associations with IPV, unintended pregnancy, contraceptive non-adherence, and poor sexual and reproductive health. In brief, the evidence suggests that:

- RCA is common and comprises a spectrum of behaviours such as pressure, manipulation, emotional blackmail, trickery, threats, and the use of physical violence [2, 7];
- The lifetime prevalence of RCA ranges between 8 and 30% depending on the sample and setting [7]. More recent studies also sit within this range (e.g. Swan and colleagues [14]; Grace and colleagues [15]);
- RCA is associated consistently with IPV, unintended pregnancy, and contraceptive non-adherence [7]. More recent work using multi-dimensional tools to measure IPV suggests that RCA is associated with more severe forms of violence [15];
- Risk factors reported for RCA include being of non-White background [15], being young [7], and being single/non-partnered or experiencing relationship issues [7, 15].

## Issues with the existing evidence base

We outline below a number of key issues which—in our view—hamper the field of RCA research and call into question some of the findings reported in the extant literature. Some of these are based on our own qualitative work in the field [16–21], whilst others are our reflections on the *lack* of empirical evidence.

### Ambiguity of the term “reproductive coercion”

Elsewhere, [17] we have argued that the term “reproductive coercion”—which is the predominant term referred to in the literature—is problematic. Although the term does accurately reflect the psychological tactics often engaged in by perpetrators, it also permits over-inclusivity. Coercion can be understood in a variety of ways, and has, on occasion, been interpreted as anything that could possibly have an impact on women’s reproductive choices. For instance, Dejoy [22] argues in her essay on structural violence that: “When state policies make contraception and abortion care inaccessible to some people, they are, in effect, replicating reproductive coercion on a structural level.” (p.45).

Although we do not deny that structural inequalities can be experienced as violence, it is not helpful to consider these sorts of external or contextual issues as being one and the same as abuse from one’s partner or close family member. Structural problems such as poor access to abortion and governmental policies that disadvantage women certainly *contribute* towards a climate where RCA can flourish, yet, they are not in themselves “reproductive coercion”. In other words, the propensity to describe RCA as a “continuum” [23] that ranges from interpersonal relationship dynamics through to government policy decisions is not evidence-based and ought to be interrogated with greater rigour.

Similarly, studies have referred to forced fatherhood experienced by men as being “reproductive coercion” [24], which again, unnecessarily obfuscates the phenomenon in question. While tricking someone into becoming a father against their will is undoubtedly problematic and potentially harmful, the context of this experience is completely different to what the research says about RCA in women. In particular, we refer here to the elements of fear and control. For women, their bodies are held hostage by a partner or family member through fear that they will be harmed physically, psychologically, financially or sexually if they do not comply. Legal explorations of coercion as a concept make it clear that the term means being forced to do something *under threat of negative consequences that will disadvantage or harm* [25]*,* yet this important aspect seems to get lost when the term is used in practice. Returning to the case of forced fatherhood, we agree that it does take an important decision—one that happens to be related to reproduction—out of a man’s hands, yet realistically we cannot argue that men are being “abused” just because a child exists that shares their DNA.

On the other hand, an issue that is clearly coercive but is often neglected within the remit of RCA is forced sterilization by family members—typically of women with disabilities. Although this behaviour can occur with “good” intentions [26, 27]—ostensibly to protect vulnerable women from unwanted pregnancies due to sexual assault or to manage menstruation—it is nonetheless an attempt to control bodily and reproductive autonomy. We do not wish to venture too much into this territory, since it engages with issues that are beyond the remit of this commentary, however, recent literature [27, 28] supports an understanding of this behaviour as a form of abuse. We recommend further investigation of this area to improve our understanding.

We also acknowledge that forced sterilisation, forced abortion and forced pregnancy have historically been perpetrated (and in some cases, still are) against women from racialized or marginalised groups. For example, studies report these behaviours in the context of colonisation [29, 30], eugenics and genocide [31], as well as being forms of gender-based violence. These state-sanctioned tactics, however, may be driven by different motives to those of the individual perpetrator. For example, Sifris [32] describes involuntary sterilisation as being primarily motivated by intersectional discrimination, whereas we typically understand violence in heterosexual relationships as being a product of male entitlement, fear and control [33] (acknowledging that, for some, the experience of IPV is also intersectional). As with the case of women with disabilities, however, we suggest that this is an issue that also merits further research to determine whether it ought to be considered within the same category as interpersonal abuse.

Lastly, we argue that referring to “reproductive coercion” obscures the fact that perpetrator tactics—such as the use of physical violence to induce miscarriage or threats to kill or harm existing children if a woman has an abortion—venture well beyond the realms of “coercion” and into abuse and violence. Consider for example the below quote from a recent study by Grace and colleagues [34] with Latina women in the US:

*He made me abort by kicking me. After he hit me, the very next minute I started to have contractions in my spine. And then I started to bleed... blood gushed out of me. And then he took me to the doctor, and they did the curettage... (p.4)*

As health researchers working with women and practitioners we have often heard participants express confusion about the term, thinking that physical or sexual violence are not within its remit [20]. Some researchers have attempted to address these issues by referring to “reproductive control”, with reproductive coercion as a subset of psychological behaviours [7]. This certainly has merit, however, again the word “control” does not necessarily encompass circumstances in which fear is present. We have also seen it referred to as “non-consensual insemination” [35], which also fails to acknowledge the abusive nature of the behaviour and does not encompass forced abortion. In our own work we have previously suggested “reproductive coercion and abuse” (RCA) as an alternative [17, 18]; this clearly positions the phenomenon as abusive while also allowing space for behaviours that are more subtle to be included. Yet, it is also somewhat unwieldy. For the remainder of this commentary we will refer to RCA, however, we welcome suggestions from other researchers and practitioners working in this area around how to more accurately and clearly refer to this form of violence against women.

### Failure to focus on intent as central

Another important consideration in understanding RCA is the issue of *intent.* By this we mean that in order to qualify as RCA, a behaviour has to be perpetrated with the intention of either impregnating a woman or preventing her from becoming or remaining pregnant. In other words, behaviours that *incidentally* have reproductive impacts ought not be included. This is best illustrated through examining the issue of stealthing (non-consensual condom removal during sexual intercourse). Given that RCA typically includes “condom sabotage” as one of its forms, some researchers have included stealthing as an example of this behaviour [36]. In many ways, this makes perfect sense. However, the removal of a condom during sex can be performed for a variety of reasons, most of which have nothing to do with reproduction. For instance, research points to loss of pleasure/sensation as a prime motivator of men’s condom “non-compliance” [37]. Brodsky, in her examination of men’s online conversations about stealthing, argues that perpetrators are motivated by a desire to force a woman to “take the guy’s load”, not as a means of reproduction but as a symbol of power and male dominance [38]. Similarly, we have argued elsewhere [16] (based on an analysis of women’s stories about their experiences) that stealthing is characterised by disrespect and selfishness whereas RCA is about intent and control. It is therefore more accurate in many cases to describe stealthing as a form of sexual violence rather than RCA.

Similarly, the issue of female genital mutilation (FGM) could be considered by some to be a form of RCA, since it impacts women’s sexual and reproductive autonomy [39] and is associated with intimate partner violence [40]. However, literature [41] suggests that FGM is undertaken primarily to control women’s *sexuality*—with reproductive outcomes being a secondary side effect, which would place it as a form of family or sexual violence rather than RCA.

Katz and colleagues [10] have argued that the perpetrator’s intent is irrelevant, and that measures ought to contain only behaviourally-specific items. Indeed, they went so far as to remove the “in order to promote pregnancy” from the reproductive coercion measures developed by Miller and colleagues when conducting their survey. Their rationale is that RCA should follow the example of IPV research, where the majority of measures focus on behaviours without mention of intent [10]. However, IPV research avoids mention of intent specifically because it can be seen as excusing the perpetrator’s behaviour when intent is not present (i.e.: if the perpetrator did not “mean” to cause harm then it is not IPV). This is not the case for RCA, where the choice is between defining a behaviour as RCA versus another form of violence and harm is present either way.

One reason why it is important to focus on intent is to improve the quality of the evidence base around RCA. The lack of clarity around intent may explain why prevalence rates for RCA vary so widely across existing research [7]. In some studies, for instance, RCA measures specify that condom removal must be for the purpose of getting a woman pregnant, whereas in others it simply asks whether women have had a partner “tamper with or remove a condom during sex” (e.g. Black et al. [36]). Obviously, asking the latter is more likely to result in a higher prevalence rate, especially amongst the younger age group who may be engaging in more casual relationships and “hook-ups” and be more at risk of stealthing. In fact, in Katz’s study [10] on college students, the lifetime prevalence rate of 30% is the highest reported in the literature. This is precisely because by removing the intentional element from survey measures they are in fact reporting a mixture of RCA, sexual violence and IPV and thus potentially falsely elevating the prevalence.

Data on risk factors and associations may also be skewed if we do not define exactly what is meant by RCA as opposed to sexual violence or IPV. For example, a recent paper reporting on associations between race/ethnicity and experiences of “reproductive coercion” suggested that Black and Hispanic women were at greater risk than white women [42]. However, the study drew on data from the US National Intimate Partner and Domestic Violence Survey where RCA is defined by two questions, one of which is whether a partner has “refused to wear a condom when you wanted them to wear one”. Given that the question does not specify that the condom refusal was to cause pregnancy, it is unclear whether the risk for Black and Hispanic women relates to RCA or sexual violence. Similarly, the authors’ finding that RCA can occur without any other type of IPV is called into question, since it is far more likely that *stealthing* rather than RCA is not associated with IPV [16]. Although the authors do acknowledge the limitations around how RCA is measured in the NIDVS, the ongoing use of RCA data from this particular survey (from a large representative community sample) by many researchers in the field continues to be problematic.

A similar problem occurs in a recent study by Grace and colleagues that used a survey to examine the prevalence and correlates for RCA amongst college students [15]. The study measured RCA using one question about contraceptive sabotage and another that asked “Have you prevented a pregnancy by using emergency contraception or ended a pregnancy using other methods, and did not tell your partner about it because you were afraid of your partner?” Arguably this question does not measure RCA at all, but rather, measures IPV.

### Insufficient exploration of the nuances between pregnancy preventing and pregnancy promoting behaviour

There are currently few studies where analyses of RCA take into account whether the perpetrator’s behaviour was pregnancy promoting or pregnancy preventing. Some commonly-used measures of RCA, such as the Reproductive Coercion Scale [43], do not even cover the full spectrum of behaviours (items asking about forced abortion are not included). Although there is not yet an empirical basis to support the theory that the context of RCA differs by type (pregnancy promoting vs pregnancy preventing), it is logical to think there may be some variance. For instance, studies report that perpetrators can seek to promote pregnancy in the hope that it will create a permanent connection to their partner [7, 44]. On the other hand, forced or coerced abortion can occur after an unintended pregnancy, where the male partner seeks to prioritise his own needs and wishes over those of his partner [7]. Qualitative work by Buchanan and Humphreys [45] similarly suggests different ways that coercive control can play out in the context of pregnancy promoting or preventing behaviours. It should also be acknowledged that both types of RCA can occur simultaneously, although little is known about the circumstances under which this occurs.

Although Miller and colleagues defined these different aspects of RCA in their ground-breaking work [1], subsequent research has paid little attention to the nuances of each of the types and RCA is treated as a homogenous phenomenon. Yet, there is a critical need for a more complex understanding of how RCA intersects with both IPV and sexual violence, as well as its association with other health issues.

## A proposed taxonomy of reproductive coercion and abuse

**Fig. 1.**
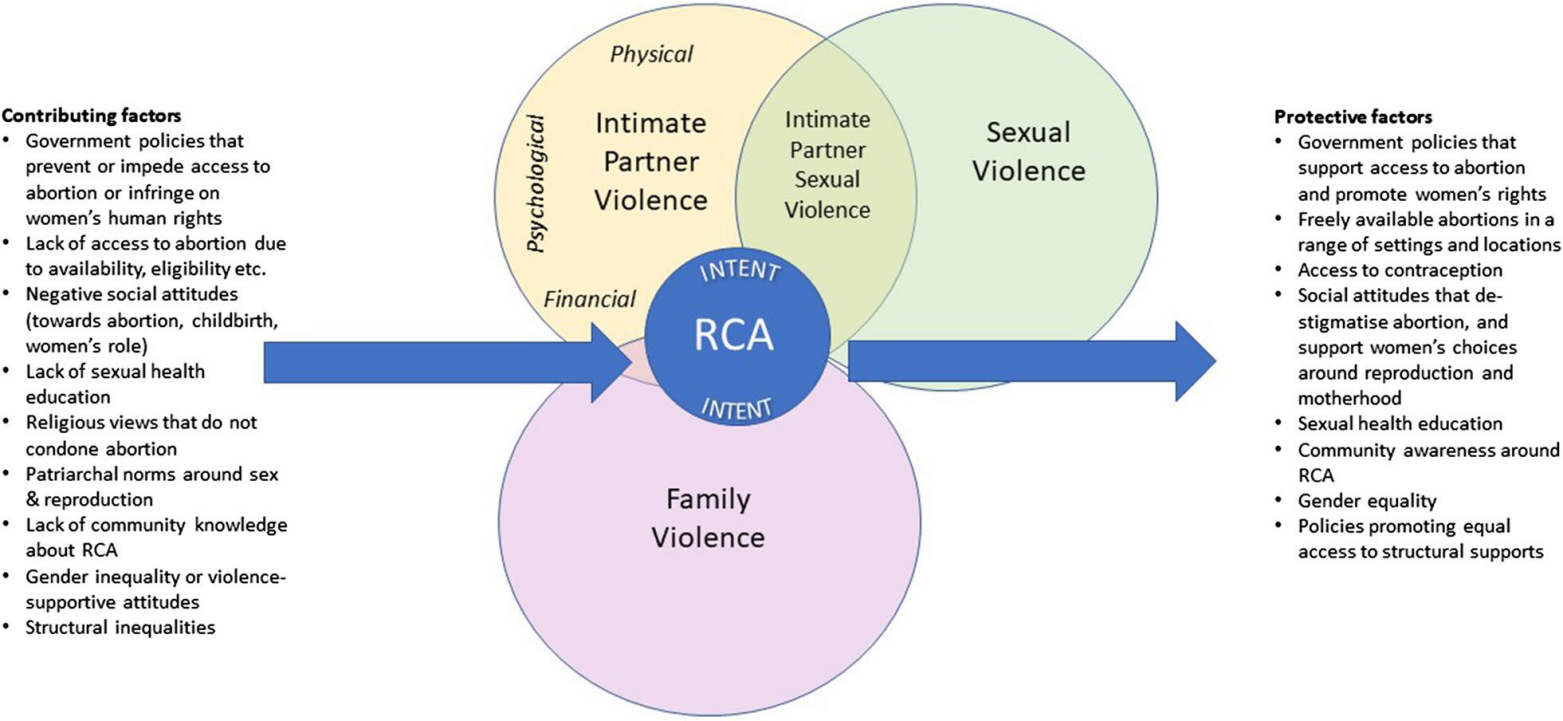
Intersections between RCA, intimate partner violence, family violence and sexual violence [21]

Drawing on recent qualitative work with practitioners, we have previously suggested that RCA sits within the intersection of sexual violence, family violence, and intimate partner violence (see Fig. 1).

Building on this framework, and given the issues discussed in the previous sections, we suggest below a classification for different behaviours that may have reproductive outcomes for an individual woman (see Table 1). Separating out behaviours in this way highlights the importance of intent to prevent or promote pregnancy (insofar as it can be established) *as well as* fear or control or both. We have taken a slightly broader view of “fear” here to encompass not just fear for personal safety but also fear of negative repercussions (e.g. partner ending the relationship, financial burden).

**Table 1:**
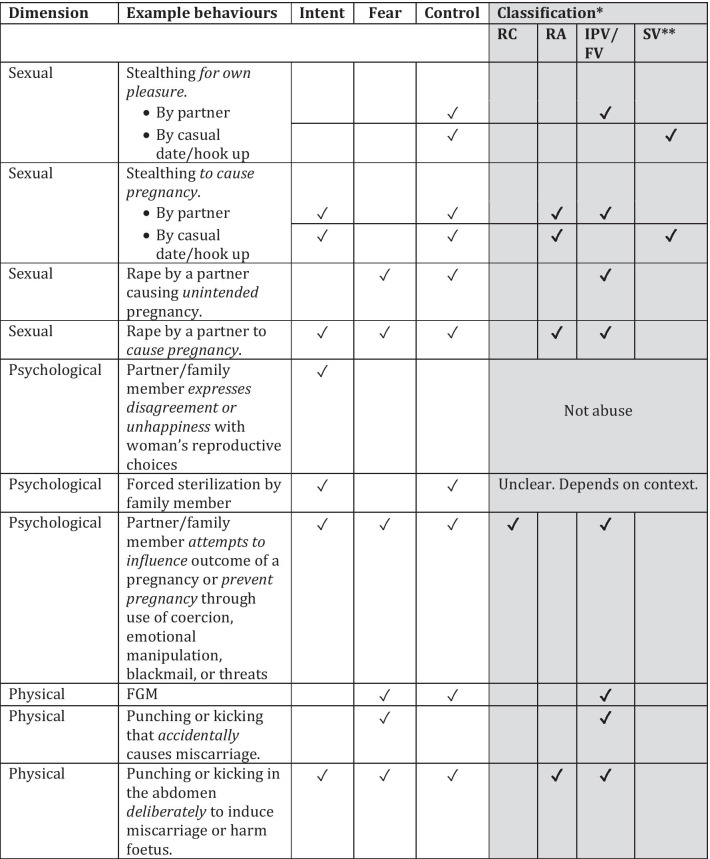
Behaviours, intent, impact and classifications

*RA* reproductive abuse, *RC* reproductive coercion, *IPV* intimate partner violence (including sexual violence), *FV* family violence, *SV* sexual violence by someone other than a partner/family member

^a^Because of the overlaps between sexual violence and intimate partner violence, we have included sexual violence by a partner under the classification of IPV

As the table shows, RCA and intimate partner violence (including intimate partner sexual violence) or family violence are intrinsically entwined. Rather than RCA being a form of intimate partner or family violence as some researchers have suggested, we propose that intimate partner or family violence is the *mechanism* by which RCA is perpetrated. In other words, RCA is physical, psychological or sexual violence harnessed for the express intent of promoting or preventing pregnancy. In particular, if we remove stealthing by casual dates or hook-ups from the list of behaviours (since the use of stealthing as a way of promoting pregnancy outside of a relationship would be highly unlikely given the lack of control a perpetrator would have over the woman), there is no remaining overlap between RCA and sexual violence outside of the relationship context.

We also propose that RCA be viewed as a continuum (see Table 2) that separates out RCA for the purpose of abortion and RCA to promote pregnancy.

**Table 2:** Behaviour matrix, reproductive coercion and abuse

	Pregnancy-promoting behaviours (examples)	Pregnancy-preventing behaviours (examples)
Coercive behaviour that attempts to influence or control women’s reproductive choices	Sabotaging contraception for the purpose of promoting pregnancy; incessant emotional pressure for a woman to be pregnant or continue a pregnancy	Emotional blackmail, threats, or other coercion to force women to carry out a termination or to use contraception to prevent pregnancy
Use of psychological or physical force to influence or control women’s reproductive choices	Threats to harm a woman or her children if she does not get pregnant or tries to prevent pregnancy	Forced sterilization of women with disabilities; threats to harm a woman or her children/family if she does not have a termination
Use of physical violence to force a woman to comply with perpetrator’s wishes	Physical violence if a woman tries to use contraception, or if a woman refuses to have sex when there is a risk of impregnation	Use of physical violence to induce miscarriage; physical violence to force a woman to use contraception
Use of sexual violence to force a woman to comply with perpetrator’s wishes	Forced sex to cause pregnancy	

**Fig. 2.**
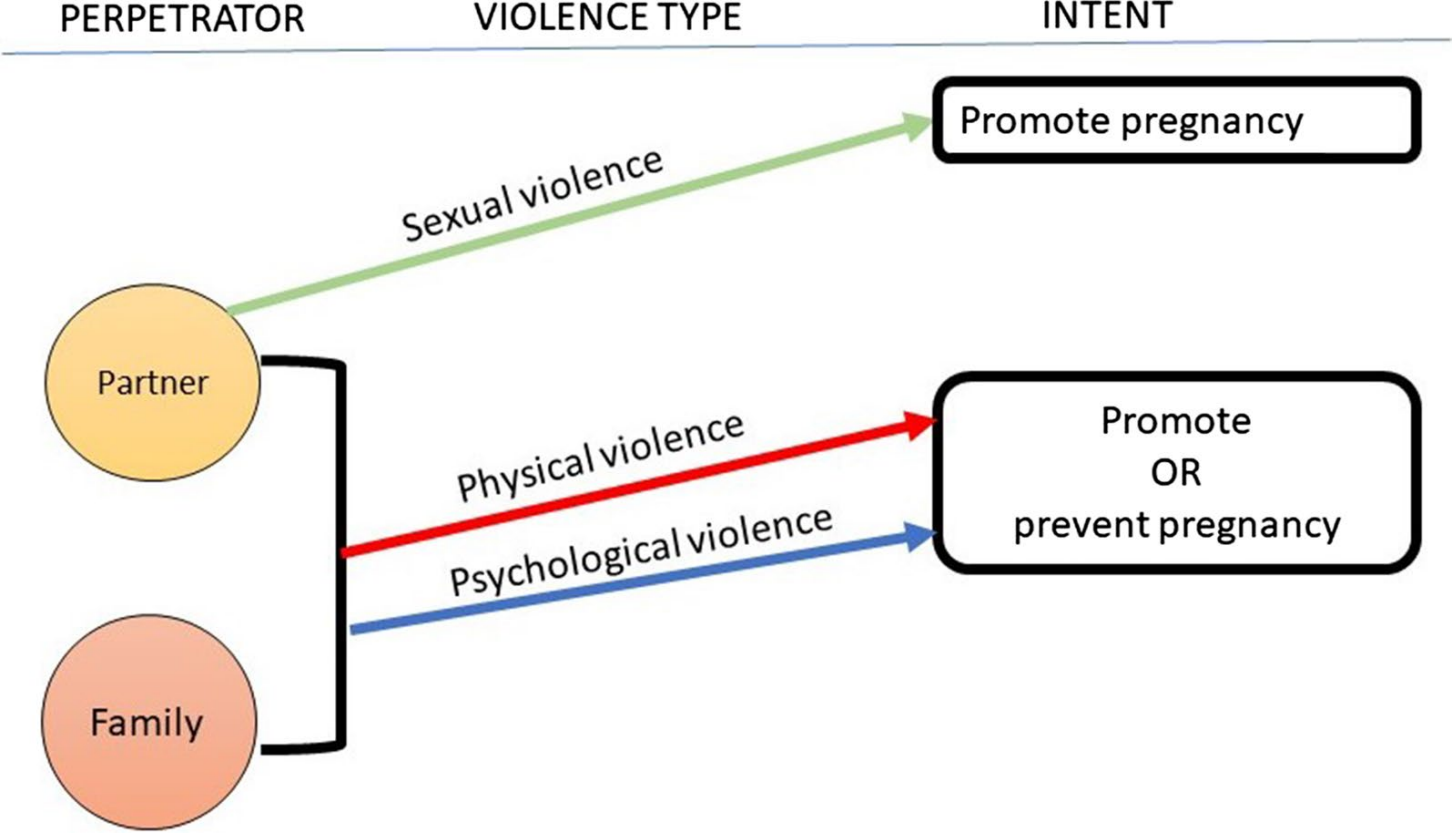
How family and intimate partner violence are harnessed to perpetrate RCA

With all of this in mind, we could consider the relationship between RCA and intimate partner or family violence to look something like this (Fig. 2):

## Recommendations for research

Based on the issues discussed in this commentary, we make the following recommendations for researchers working in this area:

- Improve conceptual clarity around RCA—We strongly suggest that if researchers want to collect meaningful and accurate prevalence and risk factor data on RCA, it is essential that measures clarify the *intention* of the behaviour rather than just the *outcome*. In doing this, we acknowledge that women may not always know or understand the motivations of the perpetrator. Measures may need to take this into account by providing options for when the intent was unclear. As a specific example, measures about nonconsensual condom removal or stealthing ought to specify that the behavior was perpetrated for the express purpose of impregnating the woman.
- Related to this, the development and evaluation of interventions addressing RCA needs to be targeted to its specific dynamics and context. In particular, the conceptual definition of RCA has implications for measuring the effectiveness of interventions.
- Greater acknowledgement of the nuances of RCA—It is important to distinguish between pregnancy promoting and pregnancy preventing behaviours in measurement of RCA so that the associations between each form of RCA and other outcomes can be properly assessed.
- Use of appropriate language that captures the abusive nature of RCA and the myriad forms of physical, psychological and sexual violence that are harnessed in order to perpetrate it. This will ensure that the elements of fear and/or control remain front and center in understanding RCA.
- Differentiation between structural/systemic issues that facilitate RCA and RCA itself. Similarly, differentiation between RCA perpetrated in an interpersonal context and reproductive violence against women in the context of conflict or colonization is also important. RCA as it is commonly understood is a form of interpersonal violence; the particular dynamics of wartime violence against women or racialized oppression are, in our view, different. We cannot simultaneously address the drivers of both with the same suite of interventions and policies.
- Research that addresses the motivations of perpetrators of RCA in order to better understand the role of intent.

Capturing all of this complexity is challenging. As a starting point, we suggest that researchers use an updated definition that builds on Miller’s work [1], but centres the elements of intent, fear and control: “Reproductive coercion and abuse is defined as any *deliberate* attempt to dictate a woman’s reproductive choices or interfere with her reproductive autonomy. It can include physical, psychological or sexual tactics and occurs in a context of fear and/or control in an interpersonal relationship.”

## Implications for health policy and practice

In terms of healthcare and service delivery, there are also important reasons to ensure that there is conceptual clarity around RCA. Although there are many elements of best practice that are applicable to all women experiencing violence [46], there are additional strategies that service providers can undertake to support women experiencing RCA [18, 19]. For example, women who have been stealthed can usually safely be offered woman-led forms of contraception such as pills or implants and may appreciate referrals to sexual assault services. On the other hand, women experiencing RCA in the context of an abusive interpersonal relationship may require assistance with safety planning and be guided towards non-detectable methods of birth control or methods that a partner cannot tamper with (e.g. injectable contraceptives). We acknowledge the argument of Katz and colleagues that women experience reproductive harm regardless of intent [10], however, our research with health practitioners suggests that a lack of shared understanding of RCA can lead to confusion around whose role it is to address it in practice, and what actions can be taken to support women [17, 18]. This has obvious implications for the quality of care that women receive.

## Conclusions

The lack of conceptual clarity around RCA has impacted upon the quality of the evidence, particularly around prevalence and risk factors. We argue that greater reflection and consensus on some of the issues raised in this commentary could help address this problem. Beyond this, there are critical gaps in the evidence base that have been neglected and require urgent attention. The most important of these is—in our view—to understand the motivations of men who perpetrate RCA. This would enable us to explore the role of intent and its relevance to the dynamics of RCA. We also need to unpack the prevalence and risk factors of different types of RCA (pregnancy promoting versus pregnancy preventing) and their associations with other types of violence, as well as exploring RCA in other settings beyond family planning. This work would strengthen the evidence-base from which to develop sound policies and best practice guidelines to better support women experiencing this hidden form of violence.

## Data Availability

Not applicable.

## Abbreviations

RCA: Reproductive coercion and abuse
SV: Sexual violence
IPV: Intimate partner violence
